# Comparison of axial length and anterior segment parameters of patients with myopia measured using 2 fourier-domain optical coherent biometry devices

**DOI:** 10.1186/s12886-024-03546-y

**Published:** 2024-07-16

**Authors:** Bingqing Sun, Yuhao Ye, Jing Zhao, Xingtao Zhou, Lingling Niu

**Affiliations:** 1 Eye Institute and Department of Ophthalmology, Eye and ENT Hospital, Fudan University, No. 19 Baoqing Road, Shanghai, 200031 China; 2 NHC Key laboratory of Myopia and Related Eye Diseases, Chinese Academy of Medical Sciences, Shanghai, China; 3 Shanghai Research Centre of Ophthalmology and Optometry, Shanghai, China; 4 Shanghai Engineering Research Center of Laser and Autostereoscopic 3D for Vision Care, Shanghai, China

**Keywords:** Axial length, Intraocular lens, Optical coherence tomography, Corneal thickness, White-to-white, Lens thickness

## Abstract

**Background:**

This study assessed the agreement of ocular parameters of patients with myopia measured using Colombo intraocular lens (IOL) 2 and IOLMaster 700.

**Methods:**

Eighty patients (male, 22; average age, 29.14 ± 7.36 years) with myopia (159 eyes) were included in this study in May 2023. The participants’ axial length (AXL), central corneal thickness (CCT), lens thickness (LT), white-to-white distance (WTW), front flat (K1), steep (K2), mean (Km) corneal keratometry, astigmatism (Astig), J0 vector, and J45 vector were measured using the IOLMaster 700 and Colombo IOL 2. The measurements from both devices were compared using the generalized estimating equation, correlation analysis, and Bland-Altman plots.

**Results:**

With the Colombo IOL 2, lower values for K2 and J0 (odds ratio [OR] = 0.587, *p* = 0.033; OR = 0.779, *p* < 0.0001, respectively), and larger values for WTW, Astig, and J45 (OR = 1.277, OR = 1.482, OR = 1.1, all *p* < 0.0001) were obtained. All ocular measurements by both instruments showed positive correlations, with AXL demonstrating the strongest correlation (*r* = 0.9996, *p* < 0.0001). The intraclass correlation coefficients for AXL and CCT measured by both instruments was 0.999 and 0.988 (both *p* < 0.0001), and Bland-Altman plot showed 95% limits of agreement (LoA) of -0.078 to 0.11 mm and − 9.989 to 13.486 μm, respectively. The maximum absolute 95% LoA for LT, WTW, K1, K2, and J0 were relatively high, achieving 0.829 mm, 0.717 mm, 0.983 D, 0.948 D, and 0.632 D, respectively.

**Conclusions:**

In young patients with myopia, CCT and AXL measurements obtained with the Colombo IOL 2 and IOLMaster 700 were comparable. However, WTW, LT, corneal refractive power, and astigmatism values could not be used interchangeably in clinical practice.

**Supplementary Information:**

The online version contains supplementary material available at 10.1186/s12886-024-03546-y.

## Background

Biometric measurements of ocular parameters are of considerable importance for the diagnosis and treatment of ophthalmic diseases [1]. Owing to myopia becoming a global public health concern, [2, 3] the assessment of axial length (AXL) in adolescents is particularly crucial. AXL changes are considered significant factors in myopia progression. For each 1-mm increase in AXL, myopia increases by approximately − 2.50 D on average [4]. Consequently, AXL can be used to predict changes in refractive error and myopia progression, [5] especially in patients under orthokeratology treatment. Furthermore, other ocular biometric parameters, such as corneal curvature, play crucial roles in fitting contact lenses and planning myopic refractive surgeries [1, 6, 7]. The selection of the size of implantable collamer lenses (ICL) also relies on precise measurements of ocular parameters such as anterior chamber depth (ACD), lens thickness (LT), and white-to-white distance (WTW) [8]. Studies have explored effective myopia prevention methods by demonstrating the correlation between AXL and ocular biometric characteristics [9, 10]. Zhang et al. [10] found that among children aged 7 to 14 years, the incidence of myopia, AXL, and ACD increase with age. Therefore, during the period of rapid physical development, the refraction and AXL should be monitored.

Since 1999, optical biometry has emerged as the conventional method for measuring AXL owing to its non-invasiveness and comparable results to those obtained through ultrasound biometry [11, 12]. Until 2009, the intraocular lens (IOL) Master 500, based on partial coherence interferometry, was the only device to measure AXL optically [13]. In recent years, the IOLMaster 700 biometer has been introduced and widely adopted. It is based on the principle of swept-source optical coherence tomography (SS-OCT), enabling visualization of the complete cross-section of the eye. Poor fixation during measurements can be monitored through macular imaging [14]. 

The Colombo IOL 2 optical biometer is a novel biometer based on spectral-domain optical coherence tomography (SD-OCT) principles. No prior research has reported its use in measuring ocular parameters in clinical settings. Therefore, this study compared ocular measurements using the Colombo IOL 2 and IOLMaster 700 in young adults with myopia. The objective was to assess the clinical value of the new Colombo IOL 2 and determine if these two devices can be used interchangeably in clinical practice.

## Methods

### Participants

This case-series study, conducted in May 2023, enrolled 80 healthy patients with myopia (159 eyes) who were scheduled for refractive surgery or received eye examination at the Eye and ENT Hospital of Fudan University. All participants underwent routine ophthalmic examinations, including slit-lamp microscopy, uncorrected visual acuity, best-corrected visual acuity, and fundus examination.

The inclusion criteria were age ≥ 18 years, no use of contact lenses for at least 2 weeks and rigid gas-permeable contact lenses for at least 4 weeks. The exclusion criteria were history of ocular surgery, progressive corneal disorders, cataracts, glaucoma, uveitis, and diabetic retinopathy.

This study was designed according to the principles outlined in the Declaration of Helsinki. It was approved by the Ethics Committee of the Eye and ENT Hospital of Fudan University (ethics code: 2020107-2). All participants provided written informed consent.

### Measurement methods

The Colombo IOL 2 (Moptim, Jiangxi, China) employs SD-OCT technology with an 850-nm measurement wavelength. The OCT image scan range covers 4 mm with an axial resolution of 5 μm. With a dual-path design, patient fixation status is confirmed through OCT images of corneal specular reflection and macular fovea to ensure precise AXL measurement.

The IOLMaster 700 biometer (Carl Zeiss AG, Jena, Germany) utilized SS-OCT technology with a 1050-nm infrared light wavelength, a scanning depth of 44 mm, and a scanning width of 6 mm. It offers an axial tissue resolution of 22 μm with a measurement speed of 2000 A-scans per second, along with six consecutive high-resolution B-scans at 0, 30, 60, 90, 120, and 150 degrees of meridians. Furthermore, its fast detection capabilities enable the simultaneous measurement of various parameters, including AXL, corneal curvature, ACD, LT, and corneal thickness. The IOLMaster 700 also had fixation confirmation, swept-source illumination, and visualized measurements [15]. 

Two skilled operators performed all the measurements for all patients using both the IOLMaster 700 and Colombo IOL 2. Measurements were conducted in a mesopic room, allowing a 5-minute adaptation period with the pupils of the patients left undilated. The examinations were conducted without a specific sequence, with a maximum time gap of 30 min between assessments using the 2 instruments. The measured parameters included AXL, central corneal thickness (CCT), LT, WTW, front corneal astigmatism (Astig), front flat corneal keratometry (K1), and front steep corneal keratometry (K2). The corneal front mean keratometry, Km, was calculated as follows: [K1 + K2]/2. The corneal astigmatism vector along the 0° meridian (J0) and 45° meridian (J45) were calculated as follows: J0 = − Astig/2×cos2α, J45 = − Astig/2×sin2α (where α is the cylinder axis). Three consecutive measurements were obtained for each eye with each device, and the mean value of each measurement was used for analysis, with the standard deviation (SD) of the three repeated measurements not exceeding 5% of the mean.

### Statistical analyses

Statistical analyses and data visualization were performed using GraphPad Prism 9 (GraphPad Software Ltd., San Diego, CA, USA) and SPSS 26.0 (IBM SPSS^®^ Statistics, Chicago, Illinois, USA). Continuous variables are described using the mean ± SD. A generalized estimating equation (GEE) approach was used to analyze the differences between the ocular measurements from the IOLMaster 700 and Colombo IOL 2. This analysis accounted for potential interference between measurements of both eyes of the same patient. Pearson’s correlation analysis was used to assess the correlations between the measurements using both instruments, and between ΔAXL (difference between AXL obtained from Colombo IOL 2 and IOLMaster 700) and AXL (obtained from IOLMaster 700). Simple linear regression models were applied for data fitting. Bland-Altman plots were used to evaluate the agreement between the two devices for various parameters. The ± 1.96 SD of the differences represented the 95% limits of agreement (LoA) for the two instruments. Intraclass correlation coefficients (ICCs) were calculated to assess the within-group correlation for different parameters. P-values less than 0.05 denoted statistical significance.

## Results

### Basic information of participants

The basic information of the participants is summarized in Table 1.

**Table 1:** Basic information of participants

	Mean	SD	Range
**Gender (n/%)**	Male (22/27.5)		
**Age (years old)**	29.14	7.36	18 to 48
**SPH (D)**	-7.00	3.54	-16.50 to -0.50
**CYL (D)**	-1.24	0.63	-2.75 to -0.25

SD, standard deviation; SPH, spherical refraction error; CYL, cylindrical refraction error

### Comparison of ocular parameter measurements from the two devices

**Fig. 1:**
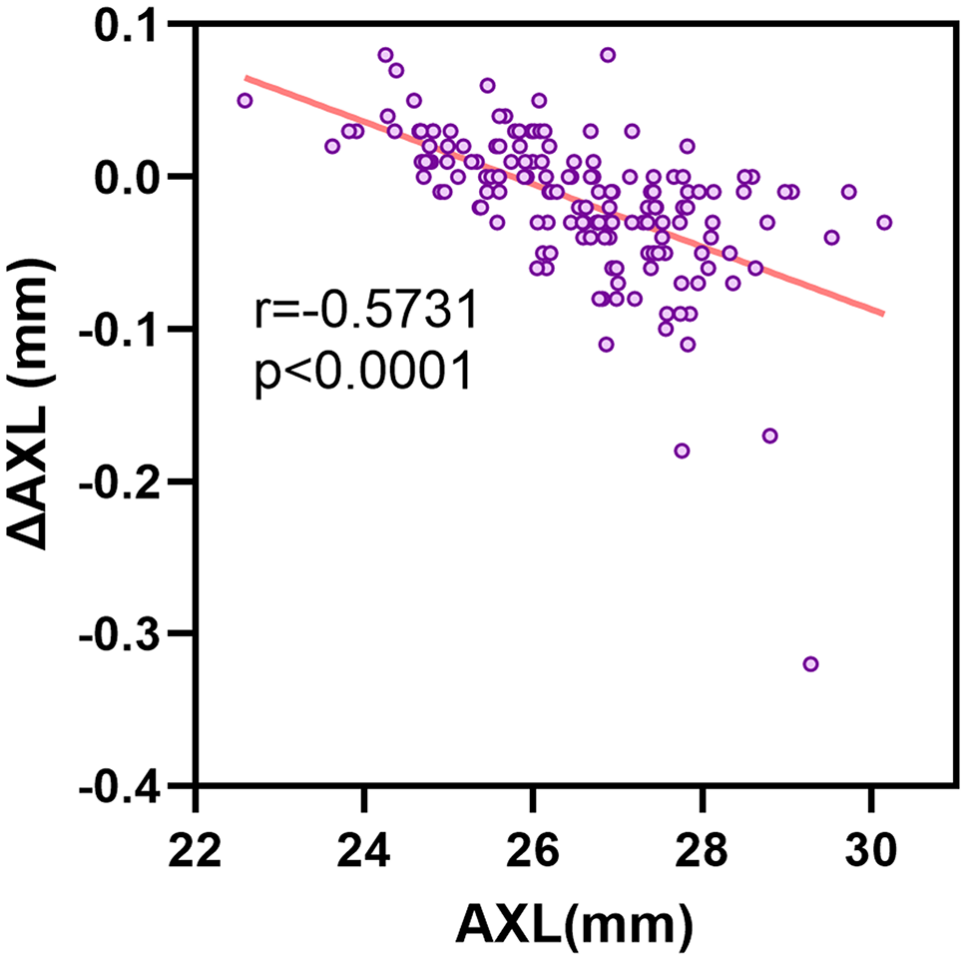
Correlation between AXL and ΔAXL. (AXL, axial length from IOLMaster 700; ΔAXL, AXL value obtained using Colombo IOL 2 – AXL value obtained using IOLMaster 700)

The ocular parameters measured using the IOLMaster 700 and Colombo IOL 2 are presented in Table 2. With the Colombo IOL 2, lower K2 and J0 (odds ratio [OR] = 0.587, *p* = 0.033; OR = 0.779, *p* < 0.0001; respectively) values were obtained, as well as higher WTW, Astig, and J45 values (OR = 1.277, *p* < 0.0001; OR = 1.482, *p* < 0.0001; OR = 1.1, *p* < 0.0001; respectively). When the patients were grouped according to AXL, the AXL measurements of two devices were not significant across all groups (all *p* > 0.05) (Table 3), while there was a negative correlation between ΔAXL and AXL (*r*=-0.5731, *p* < 0.0001) (Fig. 1).

**Table 2:** Comparison of AXL and anterior segment parameter between IOL Master 700 and Colombo IOL 2

	IOLMaster 700	Colombo IOL 2	Mean diff.	OR	*p*
**AXL (mm)**	26.54 ± 1.34	26.52 ± 1.31	-0.016 ± 0.048	0.983	0.93
**CCT (µm)**	541.1 ± 40.65	539.4 ± 38.76	-1.748 ± 5.988	0.174	0.78
**LT (mm)**	3.75 ± 0.32	3.84 ± 0.35	0.085 ± 0.212	1.065	0.291
**WTW (mm)**	11.99 ± 0.35	12.23 ± 0.40	0.244 ± 0.183	1.277	**< 0.0001**
**K1 (D)**	42.66 ± 1.45	42.50 ± 1.46	-0.151 ± 0.251	0.858	0.501
**K2 (D)**	43.97 ± 1.63	43.44 ± 1.58	-0.526 ± 0.242	0.587	**0.033**
**Km (D)**	43.32 ± 1. 50	42.70 ± 3.72	-0.339 ± 0.171	0.542	0.079
**Astig (D)**	-1.32 ± 0.68	-0.94 ± 0.52	0.39 ± 0.33	1.482	**< 0.0001**
**J0 (D)**	0.58 ± 0.40	0.33 ± 0.36	-0.248 ± 0.161	0.779	**< 0.0001**
**J45 (D)**	-0.01 ± 0.23	0.09 ± 0.20	0.095 ± 0.117	1.1	**< 0.0001**

AXL, axial length; CCT, central corneal thickness; IOL, intraocular lens; Astig, front corneal astigmatism; K1, corneal front flat keratometry; K2, corneal front steep keratometry; Km, corneal front mean keratometry; J0, corneal astigmatism vector along the 0° meridian; J45, corneal astigmatism vector along the 45° meridian; LT, lens thickness; WTW, white-to-white; Mean diff., mean difference; OR, odds ratio. The bolded p-values indicate statistical significance

**Table 3:** Differences in AXL data variability across different groups

Grouping by AXL (*n*)	IOLMaster 700	Colombo IOL 2	Mean diff.	OR	*p*
**AXL < 26 (48)**	25.08 ± 0.69	25.10 ± 0.68	0.018 ± 0.022	1.018	0.911
**26 ≤ Age < 27 (51)**	26.54 ± 0.32	26.52 ± 0.31	-0.020 ± 0.036	0.980	0.800
**Age ≥ 27 (60)**	27.94 ± 0.69	27.90 ± 0.68	-0.045 ± 0.055	0.930	0.657

AXL, axial length; Mean diff., mean difference; IOL, intraocular lens; OR, odds ratio; n, number of eyes

### Correlations between ocular parameter measurements from the two devices

**Fig. 2:**
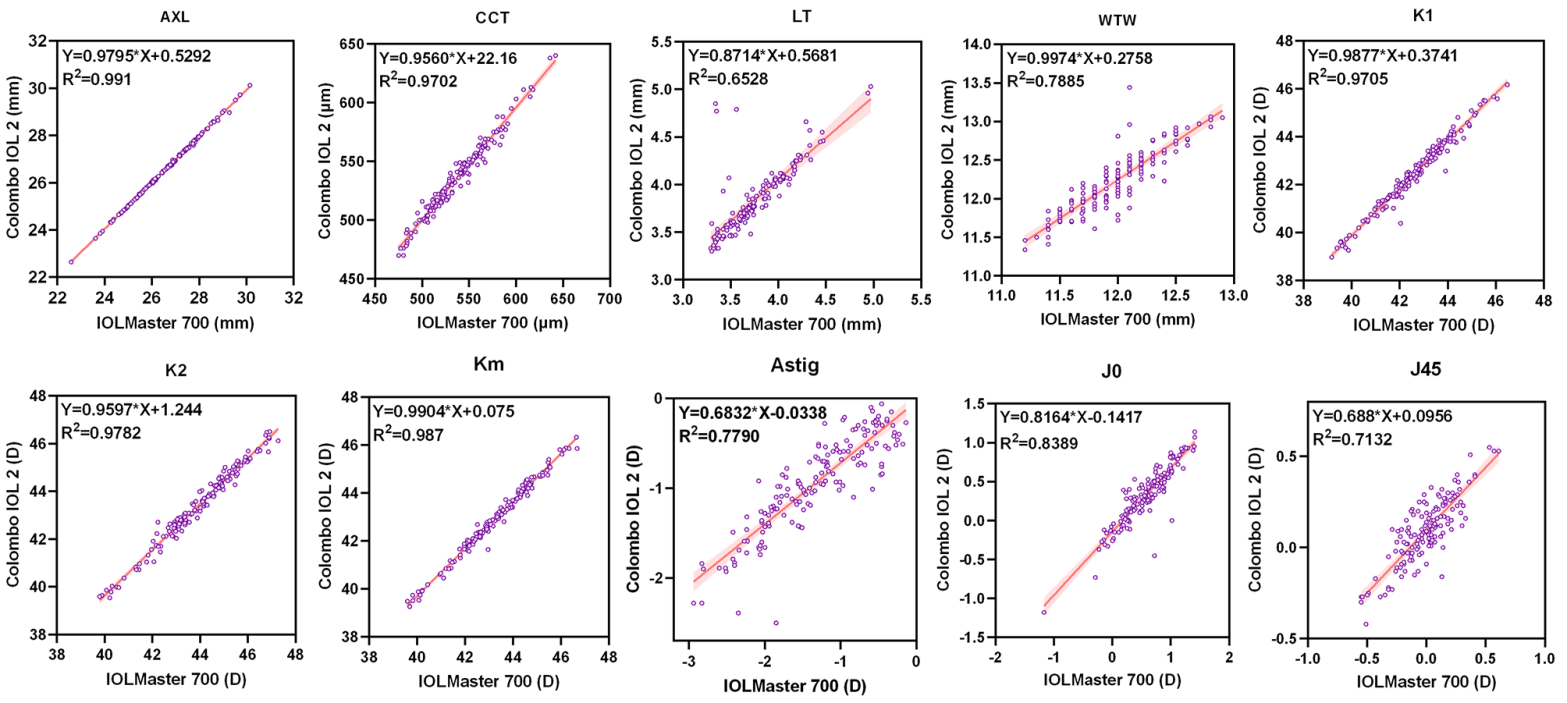
Linear regression analysis of AXL and anterior segment parameters from IOLMaster 700 and Colombo IOL 2. (AXL, axial length; CCT, central corneal thickness; IOL, intraocular lens; Astig, front corneal astigmatism; K1, corneal front flat keratometry; K2, corneal front steep keratometry; Km, corneal front mean keratometry; J0, corneal astigmatism vector along the 0° meridian; J45, corneal astigmatism vector along the 45° meridian; LT, lens thickness; WTW, white-to-white)

All measurements by two instruments showed positive correlations (all *p* < 0.0001) (Table 4). The AXL, CCT, K1, K2, Km, and J0 had strong positive correlations (all *r* > 0.9, *p* < 0.0001), with AXL demonstrating the strongest correlation (*r* = 0.9996, *p* < 0.0001). Figure 2 presents the linear regression findings.

**Table 4:** Correlations between AXL and anterior segment parameters from Colombo IOL 2 and IOL Master 700

	*r*	95% CI	*p*
**AXL (mm)**	0.9996	0.9994 to 0.9997	**< 0.0001**
**CCT (µm)**	0.990	0.9860 to 0.9925	**< 0.0001**
**LT (mm)**	0.8079	0.7433 to 0.8576	**< 0.0001**
**WTW (mm)**	0.888	0.8497 to 0.9169	**< 0.0001**
**K1 (D)**	0.985	0.9797 to 0.9891	**< 0.0001**
**K2 (D)**	0.989	0.9850 to 0.9920	**< 0.0001**
**Km (D)**	0.9935	0.9911 to 0.9952	**< 0.0001**
**Astig (D)**	0.8826	0.8423 to 0.9131	**< 0.0001**
**J0 (D)**	0.9159	0.8863 to 0.9380	**< 0.0001**
**J45 (D)**	0.8567	0.8083 to 0.8935	**< 0.0001**

AXL, axial length; CCT, central corneal thickness; CI, confidence interval; IOL, intraocular lens; Astig, front corneal astigmatism; K1, corneal front flat keratometry; K2, corneal front steep keratometry; Km, corneal front mean keratometry; J0, corneal astigmatism vector along the 0° meridian; J45, corneal astigmatism vector along the 45° meridian; LT, lens thickness; WTW, white-to-white. The bolded p-values indicate statistical significance

### Agreements of ocular parameter measurements between the two devices

**Fig. 3:**
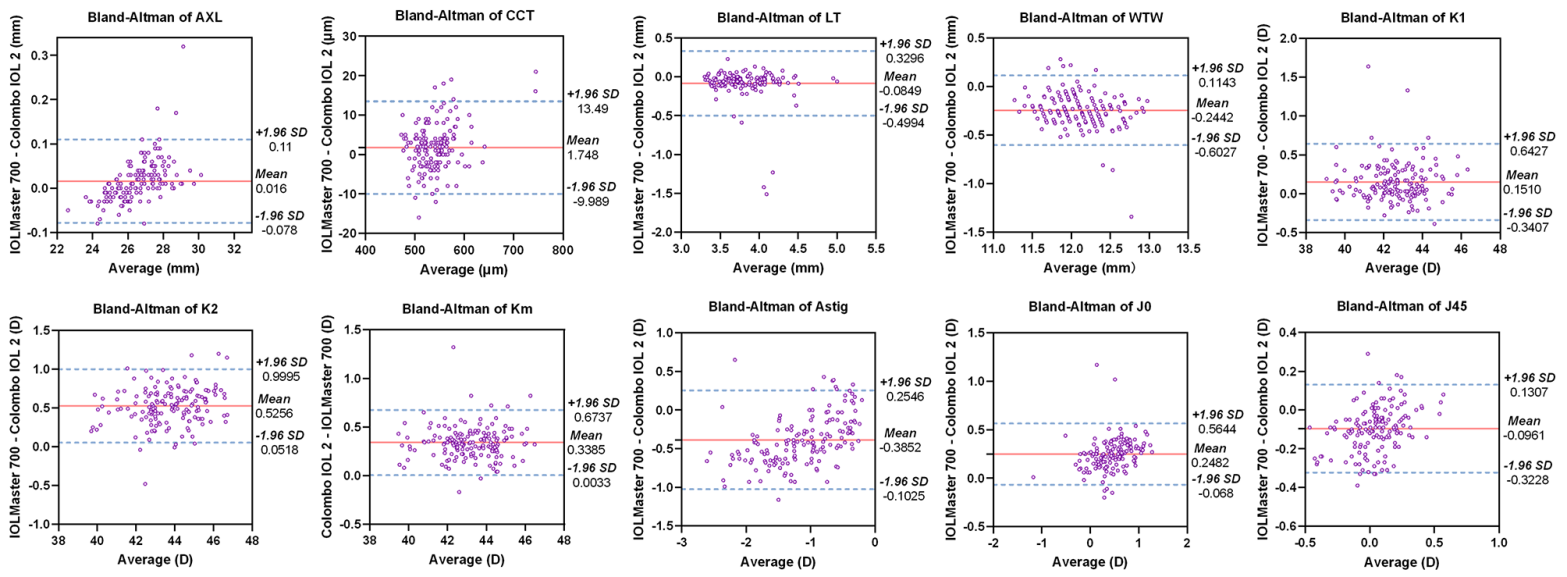
Bland-Altman plots for anterior segment parameters measured by IOLMaster 700 and Colombo IOL 2. (AXL, axial length; CCT, central corneal thickness; IOL, intraocular lens; Astig, front corneal astigmatism; K1, corneal front flat keratometry; K2, corneal front steep keratometry; Km, corneal front mean keratometry; J0, corneal astigmatism vector along the 0° meridian; J45, corneal astigmatism vector along the 45° meridian; LT, lens thickness; WTW, white-to-white; SD, standard deviation)

The ICCs for the ocular parameters measured by two instruments can be found in Table 5. The Bland-Altman analysis and graphs for all parameters are shown in Table 5; Fig. 3. The percentages of measurements for AXL, CCT, LT, WTW, K1, K2, Km, Astig, J0, and J45 falling outside 95% LoA were 3.16%, 6.29%, 3.40%, 5.06%, 3.16%, 5.70%, 4.43%, 5.81%, 3.85%, and 5.19%, respectively. The ICC for the AXLs measured using the two instruments was 0.999 (*p* < 0.0001), with the 95% LoA ranging from − 0.078 mm to 0.11 mm.

**Table 5:** Agreements between AXL and anterior segment parameters from IOL Master 700 and Colombo IOL 2

	ICC			Bland-Altman		
	ICC value	95% CI	*p*	Lower limit	Upper limit	*p*
**AXL (mm)**	0.999	0.999 to 1.000	**< 0.0001**	-0.078	0.110	**< 0.0001**
**CCT (µm)**	0.988	0.982 to 0.991	**< 0.0001**	-9.989	13.486	**0.0003**
**LT (mm)**	0.782	0.670 to 0.853	**< 0.0001**	-0.499	0.330	**< 0.0001**
**WTW (mm)**	0.729	-0.009 to 0.903	**< 0.0001**	-0.603	0.114	**< 0.0001**
**K1 (D)**	0.980	0.949 to 0.990	**< 0.0001**	-0.341	0.643	**< 0.0001**
**K2 (D)**	0.938	0.038 to 0.985	**< 0.0001**	0.052	1.000	**< 0.0001**
**Km (D)**	0.969	0.192 to 0.992	**< 0.0001**	0.003	0.674	**< 0.0001**
**Astig (D)**	0.854	0.804 to0.891	**< 0.0001**	-1.025	0.255	**< 0.0001**
**J0 (D)**	0.750	-0.042 to 0.918	**< 0.0001**	-0.068	0.564	**< 0.0001**
**J45 (D)**	0.740	0.318 to 0.877	**< 0.0001**	-0.323	0.131	**< 0.0001**

AXL, axial length; CCT, central corneal thickness; ICC, intraclass correlation coefficients; IOL, intraocular lens; Astig, front corneal astigmatism; K1, corneal front steep keratometry; K2, corneal front flat keratometry; Km, corneal front mean keratometry; J0, corneal astigmatism vector along the 0° meridian; J45, corneal astigmatism vector along the 45° meridian; LT, lens thickness; WTW, white-to-white; CI, confidence interval. The bolded p-values indicate statistical significance

## Discussion

The Colombo biometer is available in three types: the Colombo, Colombo IOL 2, and COLOMBO. Colombo IOL, which employs a wavelength of 850 nm, is primarily designed to provide refractive parameters for the prevention and control of myopia in adolescents. In this study, we used the Colombo IOL 2, which also operates at a wavelength of 850 nm. COLOMBO, which uses a wavelength of 1050 nm, is now only available in China. Both the Colombo IOL 2 and COLOMBO incorporate a myopia control module to effectively manage myopia progression and are equipped with various cataract lens formulas, enhancing preoperative planning for cataract surgery.

For patients with myopia, non-contact measurements of AXL and anterior segment parameters are crucial for detecting myopia progression and planning refractive or cataract surgeries. This study is the first in which measurements from the new Colombo IOL 2 (based on SD-OCT principles) and IOLMaster 700 (based on SS-OCT principles) are measured, with the aim of assessing the clinical utility of the Colombo IOL 2.

Through GEE analysis, the measurements of CCT using IOLMaster 700 and Colombo IOL 2 had strong positive correlations (*r* = 0.990, *p* < 0.0001), high ICCs (*r* = 0.988, *p* < 0.0001), and an average deviation of 1.748 μm according to the Bland-Altman plots (95% LoA: -9.989 to 13.486 μm). Liao et al. [16] and Cheng et al. [17] compared IOLMaster 700 with OA-2000 (also based on SS-OCT), pointing out that they had maximum absolute 95% LoA of 24.67 μm and 24.40 μm, respectively, similar to those in our study. A highly accurate corneal thickness measurement can be obtained using the Fourier-Domain OCT technology, as it can better distinguish the anterior and posterior corneal interfaces [18]. For CCT measurements, a mean deviation of 1.748 μm, as calculated using the Olsen or Kane formula, would result in a refractive power change of less than 0.005 D for artificial IOLs, [19] rendering it clinically insignificant.

Regarding corneal keratometry measurement, K2 measurements show a statistically significant difference according to GEE (OR = 0.587, *p* = 0.033). The maximum absolute 95% LoA for K1, K2, and Km measurements from the two instruments were 0.983 D, 0.948 D, and 0.670 D, respectively. Shetty et al. [20] found that the mean differences between the K1 and K2 measurements using the Anterion and IOLMaster 700, both SS-OCT biometry devices, were − 0.17 D and − 0.21 D, respectively. These values were lower than those in our study. Eibschitz-Tsimhoni et al. [21] observed that for every 1.00 D deviation in corneal keratometry, the IOL power calculation can deviate by 0.80 to 1.30 D. Therefore, the range of 95% LoA for K1 and K2 in this study suggests the need for further optimization. Similarly, the maximum absolute 95% LoA for J0 measured by both instruments was 0.632 D, greater than 0.5 D, suggesting that they cannot be used interchangeably for Astig measurement in clinical practice. Although both instruments shared similar principles in measuring anterior corneal refractive power, the IOLMaster 700 obtained 18 hexagonal reflection points within three concentric circular areas of average central 3.3-mm cornea [15], while Colombo IOL 2 obtained six reflection points within the central 2-mm cornea area, which may result in differences in determining the axis of corneal astigmatism and keratometry.

The AXL measurements using the 2 devices demonstrated a strong positive correlation (*r* = 0.9996, *p* < 0.0001), with an ICC of 0.999 (*p* < 0.0001) and an average deviation of 0.016 mm according to the Bland-Altman plot. The Colombo IOL 2 measured slightly shorter AXLs (OR = 0.983, *p* = 0.93), and as the axial length increases, the AXL measured by Colombo IOL 2 is increasingly shorter compared to IOLMaster 700 (*r*=-0.573, *p* < 0.0001). The reason is that the two instruments use different light sources and signal acquisition methods [22–25], and each has a proprietary formula to convert the optical path length to the measured AXL. As the AXL increases, the calculated differences in AXL become more pronounced. However, such difference between two instruments is insufficient to cause a clinically significant detection threshold for a 0.25 D refractive power change [4, 26]. Sorkin et al. [24] conducted a comparative study of eye parameters measured using Eyestar 900 and IOLMaster 700, two SS-OCT biometry devices, in cataract surgery patients and reported that the average difference in AXL measurements was 0.014 mm, which is acceptable in clinical practice.

A good agreement in the LT measurement between the two devices was detected (ICC value: 0.782, *p* < 0.0001). The Bland-Altman plot showed an average deviation of -0.085 mm for LT (with a 95% LOA from − 0.499 mm to 0.330 mm, *p* < 0.0001). The GEE analysis revealed that the LT measurement using the Colombo IOL 2 was slightly higher (OR = 1.065, *p* = 0.291). Fişuş et al. [27] and Oh et al. [28] found that the average differences between the LT measurements obtained using Anterion and IOLMaster 700 were 0.06 mm and − 0.0591 mm, respectively, which were slightly lower than the differences between the measurements using the Colombo IOL 2 and IOLMaster 700. The relatively lower agreement in LT measurements between these two instruments may be attributed to non-dilated pupils, which can be easily affected by fixation light brightness. Furthermore, the IOLMaster 700 employs a longer wavelength and greater penetrating power. This results in a more precise signal from the posterior lens surface, leading to deviation of LT calculations between the two instruments. Precise LT measurements are crucial for ophthalmic surgical planning. Personalized ICL size selection considers the anterior chamber structure, including the accurate measurement of LT. Additionally, LT is an essential parameter for predicting post-ICL vault height [29, 30]. 

The WTW measured using the Colombo IOL 2 was moderate in agreement (ICC value: 0.729, *p* < 0.0001) with IOL Master 700 and with significant larger readings (OR= 1.277, *p* < 0.0001). An average deviation of -0.244 mm for WTW (with a 95% LOA from − 0.603 mm to 0.114 mm, *p* < 0.0001) was also observed from the Bland-Altman plots. WTW measurements are crucial for determining ICL sizes, as WTW is used as an alternative measurement for sulcus-to-sulcus distance. Considering that the step size of ICLs is close to 0.5 mm, the differences in the WTW measurements obtained using the two devices in this study cannot be overlooked. An overestimated WTW measurement can result in the selection of an ICL size that is too small, which can lead to lens rotation, displacement, and suboptimal postoperative visual outcomes [31]. Additionally, several studies have found that WTW is an important parameter for predicting post-ICL vault height [32, 33]. The reasons for the deviations in WTW measurements include differences in how the devices position the corneal limbus. Each device uses a grayscale step to determine the point of transition between the white sclera and the darker iris. Colombo IOL 2 adopts shorter wavelength, which results in less diffraction and higher resolution, allowing more accurate identification of the transition. Moreover, WTW measured by IOLMaster 700 has a step size of 0.1 mm, while WTW measured by the Colombo IOL 2 has a step size of 0.01 mm. Other factors influencing transition identification include corneal diseases affecting corneal edge transparency, eyelash shadows, shadows from the device, and nasal shadows [34]. 

This study had several limitations. The sample size was rather small and was solely composed of patients with myopia. Healthy participants without refractive error or patients with other ocular diseases should be included in future studies. Future studies should analyze whether other Colombo biometers, such as COLOMBO with a wavelength of 1050 nm, can achieve similar discrepancies.

## Conclusions

In young patients with myopia, CCT and AXL measurements obtained with the Colombo IOL 2 and IOLMaster 700 were comparable. However, WTW, LT, corneal refractive power, and astigmatism values could not be used interchangeably in clinical practice.

### Electronic supplementary material

Below is the link to the electronic supplementary material.

Supplementary Material 1

## Data Availability

The datasets generated and/or analyzed during the current study are not publicly available due to funding requirements but are available from the corresponding author upon reasonable request.

## Abbreviations

ACD: Anterior chamber depth
AXL: Axial length
CCT: Central corneal thickness
GEE: Generalized estimating equation
ICC: Intraclass correlation coefficients
ICL: Implantable collamer lenses
IOL: Intraocular lens
J0: Corneal astigmatism vector along the 0° meridian
J45: Corneal astigmatism vector along the 45° meridian
K1: Front flat corneal keratometry
K2: Front steep corneal keratometry
K m: Front mean corneal keratometry
LoA: Limits of agreement
LT: Lens thickness
OR: Odds ratio
SD: Standard deviation
SD-OCT: Spectral-domain optical coherence tomography
SS-OCT: Swept-source optical coherence tomography
WTW: White-to-white
